# Text-mining-assisted biocuration workflows in Argo

**DOI:** 10.1093/database/bau070

**Published:** 2014-07-18

**Authors:** Rafal Rak, Riza Theresa Batista-Navarro, Andrew Rowley, Jacob Carter, Sophia Ananiadou

**Affiliations:** ^1^National Centre for Text Mining, School of Computer Science, University of Manchester, UK and ^2^Department of Computer Science, University of the Philippines Diliman, Philippines

## Abstract

Biocuration activities have been broadly categorized into the selection of relevant documents, the annotation of biological concepts of interest and identification of interactions between the concepts. Text mining has been shown to have a potential to significantly reduce the effort of biocurators in all the three activities, and various semi-automatic methodologies have been integrated into curation pipelines to support them. We investigate the suitability of Argo, a workbench for building text-mining solutions with the use of a rich graphical user interface, for the process of biocuration. Central to Argo are customizable workflows that users compose by arranging available elementary analytics to form task-specific processing units. A built-in manual annotation editor is the single most used biocuration tool of the workbench, as it allows users to create annotations directly in text, as well as modify or delete annotations created by automatic processing components. Apart from syntactic and semantic analytics, the ever-growing library of components includes several data readers and consumers that support well-established as well as emerging data interchange formats such as XMI, RDF and BioC, which facilitate the interoperability of Argo with other platforms or resources. To validate the suitability of Argo for curation activities, we participated in the BioCreative IV challenge whose purpose was to evaluate Web-based systems addressing user-defined biocuration tasks. Argo proved to have the edge over other systems in terms of flexibility of defining biocuration tasks. As expected, the versatility of the workbench inevitably lengthened the time the curators spent on learning the system before taking on the task, which may have affected the usability of Argo. The participation in the challenge gave us an opportunity to gather valuable feedback and identify areas of improvement, some of which have already been introduced.

**Database URL:** http://argo.nactem.ac.uk

## Introduction

Data curation from biomedical literature had been traditionally carried out as an entirely manual effort, in which a curator handpicks relevant documents and creates annotations for elements of interest from scratch. To increase the efficiency of this task, text-mining methodologies have been integrated into curation pipelines. In curating the Biomolecular Interaction Network Database (1), a protein–protein interaction extraction system was used and was shown to be effective in reducing the curation workload by 70% (2). Similarly, a usability study revealed that the time needed to curate FlyBase records (3) was reduced by 20% with the use of a gene mention recognizer (4). Textpresso (5), a text-mining tool that marks up biomedical entities of interest, was used to semi-automatically curate mentions of *Caenorhabditis elegans* proteins from the literature and brought about an 8-fold increase in curation efficiency (6). More recently, the series of BioCreative workshops (http://www.biocreative.org) have fostered the synergy between biocuration efforts and text-mining solutions. The user-interactive track of the latest workshop saw nine Web-based systems featuring rich graphical user interfaces designed to perform text-mining-assisted biocuration tasks. The tasks can be broadly categorized into the selection of documents for curation, the annotation of mentions of relevant biological entities in text and the annotation of interactions between biological entities (7).

In this article, we present Argo and investigate whether this primarily text-mining workbench is suitable to support the aforementioned biocuration tasks. In a previous curation effort, the automatic processing within Argo was found to be adequate for the completion of ∼84% of a drug and enzyme annotation task, the remainder of which was completed manually by a domain expert using Argo’s manual annotation editor (8). Here, we demonstrate Argo in the context of a biocuration task that was showcased in the recently completed BioCreative IV challenge. Some of the capabilities we describe were introduced as a result of the participation in the challenge. The user feedback we received allowed us to better understand the needs of biocurators and adjust our system accordingly.

In the remainder of this article, we briefly introduce the system and elaborate on specific capabilities that support text-mining-assisted biocuration activities. We then present a detailed discussion of our participation in the interactive track at BioCreative IV, in which Argo was applied to a specific biocuration task, the annotation of metabolic process concepts and report on the results obtained and insights gained from this exercise. We also provide a comparison of Argo against other biocuration systems. We conclude by summarizing the lessons learned from this study and by formulating plans for improvement to better address the requirements of biocurators.

## System overview

Argo (http://argo.nactem.ac.uk) is a multi-user Web-based workbench for collaborative development and evaluation of text-processing workflows (8). The workbench includes an ever-growing library of elementary processing components or analytics, developed mostly at the National Centre for Text Mining (NaCTeM). They range from simple data (de)serialization to natural language processing (NLP) to semantic annotation (named entity and relationship recognition). The principal features of Argo include the easy combination of elementary text-processing components to form meaningful and comprehensive processing workflows, the ability to manually intervene in the otherwise automatic process of annotation by correcting or creating new annotations and the enabling of user collaboration by providing sharing capabilities for user-owned resources. The workbench is meant to accommodate a variety of tasks and domains.

The tasks in Argo are defined by creating workflows, i.e. by arranging a selection of elementary processing components by means of interconnecting their outputs and inputs, and setting up their configuration parameters. The most common approach is an arrangement that forms a pipeline or a serial workflow. The generic flow involves reading source data, performing automatic and/or manual annotation on the data and then saving the annotations (usually together with the source data). Each of these three steps is highly configurable by the choice of elementary processing components that best fit the task at hand. For example, reading source data may be accomplished by deserializing user-uploaded files in various formats, such as plain text, XML and RDF, as well as by fetching data from remote Web services, such as search engines.

Argo is available as a Web application and thus is accessed with a Web browser. Its graphical user interface (GUI) provides access to the complete functionality of the workbench.

## User resources

**Figure 1.**
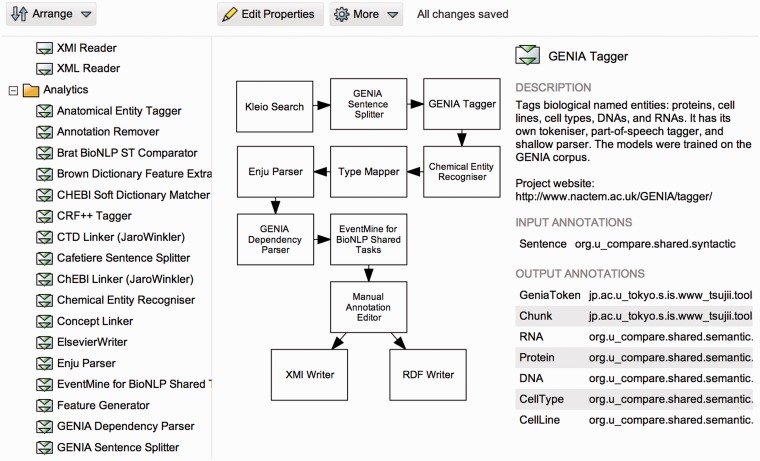
A screenshot of Argo’s workflow diagramming window. Users create their workflows graphically by selecting and placing elementary processing components onto a drawing canvas and interconnecting them to form meaningful processing units.

Users interact with Argo primarily by developing workflows and—if source data is not fetched remotely—by uploading their documents for processing with the workflows. Workflows are defined by users by visually arranging elementary components into interlinked graphs using a dedicated graphical block diagram editor shown in Figure 1. Processing components are represented in a diagram as blocks that are linked with connectors. Although the most common arrangement is a pipeline (where the output of each component is connected to at most one input of another component), Argo also supports complex arrangements with multiple branching and merging points as shown in the figure.

**Figure 2.**
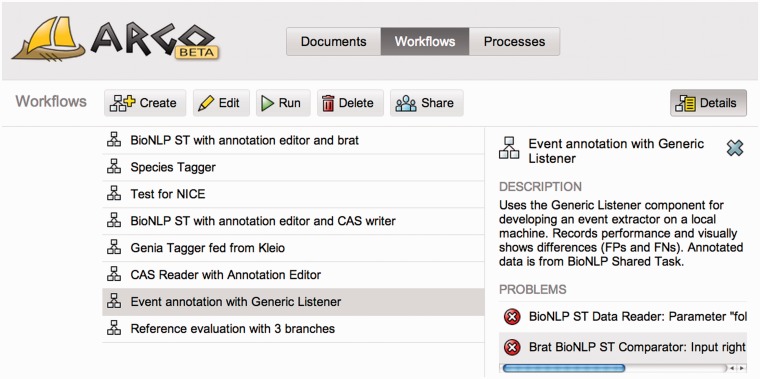
A screenshot of the Argo application showing the Workflows panel that lists and enables managing user-created workflows. The right-hand side panel provides a description of the currently selected workflow as well as warning messages informing the user of problems with the underlying components’ configuration or connections.

Workflows created by users are listed and managed in the Workflows panel (visible in Figure 2), which also allows for initiating the processing of workflows whose progress may be tracked, in turn, in the Processes panel. Users can manage their documents in the Documents panel whose functionality resembles that of a typical file system. The documents are usually expected to be text files that are used as source data for processing workflows. Analogously, the workflows may store the intermediate or final results of processing, e.g. XML files containing annotations, in the same document space, that are later available for users to download, or for use in further processing.

Both workflows and documents can be shared among the users of Argo. This makes it especially useful in terms of distributing the workload of carrying out a curation task: users with a technical background may entirely design a workflow, which they can then share with, for example, biologists for them to run the workflow and provide annotations using their domain expertise.

## Technology and interoperability

Argo supports and is based on the Unstructured Information Management Architecture (9). The architecture is an OASIS standard (http://www.oasis-open.org/committees/uima) for ensuring interoperability of individual processing components by defining common data structures and interfaces. Annotations created in this architecture must adhere to customizable and well-defined annotation schemata or type systems. Each processing component imports (and supports) one or more such type systems and is responsible for populating a common annotation structure that is passed between components in a workflow for further processing.

The actual processing of workflows is carried out on a multi-core server, with additional support for execution on high-throughput cloud computing frameworks, such as HTCondor.

## Biocuration in Argo

**Figure 3.**
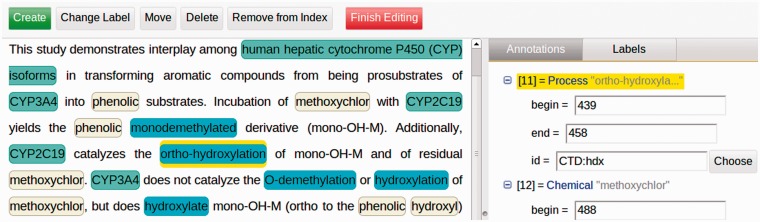
A screenshot of the fragment of Argo’s manual annotation editor. The editor allows users to visually create, modify or delete annotations in the left-hand-side panel, as well as fill in more specific information (governed by a given annotation schema) for each of the annotations in the right-hand-side panel.

**Figure 4.**
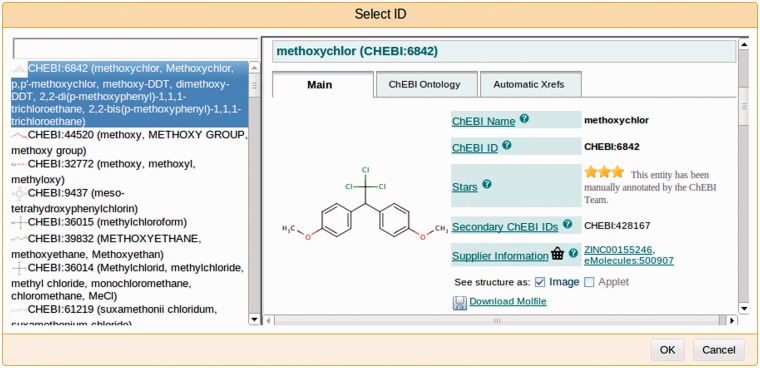
A screenshot of a window for selecting an identifier for an annotated fragment of text from an external resource—in this case, the ChEBI ontology. Users may select the most suitable entry by browsing a selection in the left-hand-side panel and viewing the details in the central panel.

Argo includes several features that render the workbench suitable for curation activities. In particular, one of the features allows users to manually intervene in the processing of a workflow by means of user-interactive processing components. An example of this type of component is the *Manual Annotation Editor*, a graphical interface that lets users view, modify or delete existing annotations, or add new ones [We use the term ‘annotation’ to denote any added information that is not part of the original data (document). They include span-of-text annotations as well as non-text-bound annotations that may contain primitive attributes (such as string, integer, boolean) as well as references to other annotations]. The editor is the primary interface between biocurators and the system. The functions of the editor, a fragment of which is shown in Figure 3, include the following:

- Selecting a document for annotation from a list of available (previously processed) documents;
- removing or reassigning labels to already (possibly automatically) annotated spans of text;
- adding new annotations by selecting a span of text and assigning a label to it from a set of available labels (the central panel in Figure 3);
- adding new or deleting existing document-level or relationship annotations that are not directly made on a span of text (i.e., meta annotations);
- editing annotation features (attributes) via an expandable tree structure (shown on the right-hand-side panel in Figure 3);
- support for overlapping (intersecting) annotations;
- a GUI for assigning identifiers from external databases (see Figure 4); and
- filtering of annotations by semantic labels.

Relationships or interactions between biological entities are constructed by first marking spans of text in the central panel shown in Figure 3 and assigning labels (types). Thereafter, the appropriate relationship annotation is created on the right-hand-side panel and the relevant entities are moved into the newly created relationship annotation. The cohesion of these structures is maintained automatically and adheres to the annotation schemata in use.

## Components

The fundamental building blocks of workflows are processing components or analytics. Argo has an ever-growing library of components whose functionalities range from data reading/writing to NLP and text-mining analytics, to annotation manipulation and evaluation. Tables 1–3 list some of the components that are of interest to biological curation tasks. Deserializers or data readers, shown in Table 1, include local file readers, generic XML and RDF readers, search engines and data-specific readers, such as the BioNLP Shared Task Reader. Analytics components shown in Table 2 are the main text-mining processing units and include automatic recognition and ontology binding of biologically relevant concepts, such as genes, chemicals and species names, as well as complex interactions (events) between these concepts. Finally, Table 3 lists utility components that support automatic processing. These include the annotation editor, a component for aligning heterogenous annotation schemata and components for evaluating workflows against a gold standard data and for evaluating inter-annotator agreement if the manual annotation of the same data is performed by more than one curator. The collection also features a component that allows users to connect their own components running on their own machine to an Argo workflow running on the server. This is especially useful in the development of dedicated processing components that are not featured in Argo.

**Table 1.** Data serialization and deserialization components available in Argo that may be used in biocuration workflows as terminal components for reading source data and saving the results of processing

Component name	Description
Document Reader	Deserializes text files stored in the user’s personal space (i.e. the Documents panel)
Kleio Search	Remotely fetches PubMed abstracts matching a query set as a parameter
PubMed Abstract Reader	Fetches abstracts directly from PubMed using a list of PubMed IDs as input
Input Text Reader	Reads text supplied in a parameter
BioNLP Shared Task Reader	Deserializes triple files (containing plain text, stand-off annotations of named entities and stand-off annotations of events or structured relationships) as defined in the BioNLP shared task^a^
XMI Reader/Writer	(De)serializes entire CASes (data and annotations) from/into the XML Metadata Interchange (XMI) format
RDF Reader/Writer	(De)serializes entire CASes from/into RDF which may then be reused in other applications, e.g. in query engines supporting SPARQL
BioC Reader/Writer	(De)serializes selected annotations from/into BioC format^b^

^a^http://2013.bionlp-st.org

^b^http://bioc.sourceforge.net

**Table 2.** Text analysis components available in Argo that may be used in biocuration workflows to produce annotations

Component name	Description
GENIA Sentence Splitter	A sentence splitter trained on biomedical text (10)
GENIA Tagger	Performs tokenization, part-of-speech and chunk tagging, and recognition of genes or gene products (e.g., proteins, DNA, RNA) (11)
GENIA Dependency Parser	A dependency parser optimized for biomedical text (12)
Enju Parser	Returns phrase and predicate-argument structures for general and biomedical text (13)
Anatomical Entity Tagger	A machine learning-based anatomical entity mention recognizer (14)
NERsuite	A named entity recognizer implemented on top of the NERsuite package.^a^ Includes several models to choose from.
Chemical Entity Recogniser	A named entity recognizer optimized for chemical text (15). Includes several models to choose from.
OscarMER	A refactored version (16) of OSCAR 3 (17), which recognizes chemical concepts (e.g. compounds, reactions) using a maximum entropy model
Species Tagger	A tagger for species names based on a dictionary look-up method (18)
CTD Linker	Normalizes an action term to one of the types in the CTD interaction types ontology.
ChEBI Linker	Normalizes a chemical compound name to an entry in the Chemical Entities of Biological Interest (ChEBI) database.
UniProt Linker	Normalizes a name of a gene or gene product to a UniProt entry.
EventMine	A machine learning-based event extractor with models for GENIA, epigenetics, infectious diseases, pathway and cancer genetics event types (19–21)

^a^http://nersuite.nlplab.org

**Table 3.** Utility components available in Argo that may be used in biocuration worfklows to support automatic processing

Component name	Description
Manual Annotation Editor	A user-interactive component that supports visualization and manipulation of annotations, allowing the user to manually intervene in the processing of a workflow
SPARQL Annotation Editor	Creates, removes and modifies annotations using a SPARQL query (22). May also be used for complex type conversions.
Generic Listener	An interface that allows a user to plug-in their own components running on a local machine (23)
Agreement Evaluator	Analyses two or more input annotation efforts (coming from different branches in a workflow) and produces a tab-separated file, reporting agreement rates between the inputs; may serve to compute inter-annotator agreement
Reference Evaluator	Compares automatically generated annotations against reference annotations and produces a tab-separated file reporting evaluation results.

## Workflows

Argo facilitates text-mining-assisted biocuration by means of task-specific highly customizable workflows. The workbench’s wide range of available components allows users to define semi-automatic curation workflows based on their specific needs.

The workflow shown in Figure 1 demonstrates how the three major elementary biocuration tasks, i.e. document selection, concept recognition and concept interaction identification, can be realized in Argo. The task is defined as the identification of interactions signifying metabolic processes in a selection of PubMed abstracts. It involves the annotation of two types of concepts in the abstracts, namely, chemical compounds and genes or gene products.

The illustrated workflow makes use of Kleio Search, a special case of a data reader component that connects to a Web service and fetches remote content matching a user-defined query. The Web service behind this particular component is a programmatic access point to the search engine Kleio (www.nactem.ac.uk/Kleio), which indexes PubMed abstracts and facilitates faceted search (24). The parameters of the component are a query string and a choice of ordering results by relevance or publication date. Each retrieved PubMed abstract is segmented into sentences and tokens by the GENIA Sentence Splitter and GENIA Tagger components, respectively. The latter also includes the recognition of several semantic types including genes and gene products (as a single type). Instances of the other concept type, namely chemicals, are identified by the Chemical Entity Recognizer. The EventMine component ingests the recognized concepts and finds interactions between them (if such exist). All the concept and interaction recognizers use machine learning models to accomplish their respective tasks. Additionally, Enju Parser and GENIA Dependency Parser support the EventMine component by producing a deep syntactic analysis of sentences. The inclusion of SPARQL Annotation Editor in the workflow is needed for aligning annotation schemata between the types of the relevant concepts and the types expected by EventMine. The user-interactive manual annotation editor allows annotators to inspect and correct (where necessary) the automatically generated annotations. Lastly, the annotations are saved as XML Metadata Interchange (XMI) files as well as widely adopted RDF graphs.

We emphasize that workflows in Argo are highly modular, allowing their customization by means of interchangeable components. For instance, in the example workflow, users may opt to replace the Kleio Search component with a Document Reader, which will then allow them to work with documents stored in their dedicated storage space that comes with each user account. Yet another variation involves using an XMI Reader that is capable of reading the results of the processing of other workflows that featured an XMI Writer as their data consumer component. Similarly, any of the syntactic and semantic analysis components may be replaced by other suitable components available in the library, as well as by users’ own solutions that can be introduced into a workflow by the Generic Listener component (described in Table 3).

## Use case: annotation of metabolic processes

To validate the suitability of Argo for biocuration tasks, we participated in the interactive track of the recently concluded BioCreative IV workshop, whose aim was to assess the state of the art in bridging text mining and biocuration. One of the requirements imposed on participating systems was that they had to feature a graphical user interface accessible as a Web application.

## Background

The curation task we proposed involved the annotation of concepts relevant to metabolic processes. One of the core disciplines of systems biology, metabolomics, the study of organic and inorganic small molecules (i.e. metabolites) (25), plays a central role in drug and biomarker discovery (26), and facilitates the advancement of research focussing on the understanding, prevention and treatment of diseases (27). To date, biochemical databases storing information on metabolites and relevant processes undergo purely manual curation. In the KEGG PATHWAY (28), Reactome (29) and Rhea (30) databases, biochemical reaction entries are manually linked to scientific documents providing evidence of the reactions. Even greater manual effort is required in the population and maintenance of SABIO-RK (31) and MetaCyc (32), which contain reaction information manually extracted from published literature. The fact that the upkeep of such databases has yet to be supported by text mining validates the previously reported observation that the curation of metabolic pathways has received little attention from the biomedical NLP community, compared with that of signalling pathways (33). This gave us the motivation to define a task that explores the semi-automatic curation of metabolic processes, as part of our participation in the BioCreative track. Having already demonstrated the automatic linking of reactions to textual evidence in previous work (34), we focussed our case study on facilitating their automatic extraction from scientific literature using Argo as the biocuration platform.

We have undertaken this case study on metabolic process concept annotation with the following objectives: (i) to measure the accuracy of automatic annotation by evaluating the performance of the underlying text-mining components in Argo against human curators, and (ii) to quantify the feasibility of semi-automatic curation in Argo by collecting feedback from the curators and by comparing time spent on text-mining-assisted annotation against that on purely manual annotation. The outcome of this study was aimed at eventually facilitating the development of methods for the semi-automatic curation of biochemical reaction databases.

## Curation task set-up

In designing the task, we follow the definition of metabolic process from the chemical–gene interaction types ontology (http://ctdbase.org/help/ixnQueryHelp.jsp#actionType) of the Comparative Toxicogenomic Database (CTD) (35), i.e. ‘the biochemical alteration of a molecule’s structure, excluding changes in expression, stability, folding, localization, splicing and transport’. Below is an example of a metabolic process expressed in scientific text:

The known activity of cytochrome P450 46A1 (P450 46A1) is 24(S)-hydroxylation of cholesterol. This reaction produces biologically active oxysterol, 24(S)-hydroxycholesterol, and is also the first step in enzymatic degradation of cholesterol in the brain.

  [Source: PubMed abstract 14640697]

Described in these two sentences is a metabolic process, specifically, the *24(S)-hydroxylation* of the compound *cholesterol* by the enzyme *cytochrome P450 46A1*, resulting to the production of *24(S)-hydroxycholesterol*. Based on the concept types frequently involved in metabolic processes, we defined the curation task as the annotation of chemical compounds (CCs), genes or gene products (GGPs) and expressions signifying metabolic processes (triggers). Both CCs and GGPs may play the role of reactant (entity undergoing the alteration), product (entity into which the reactant is changed) or modifier (entity driving the alteration) in a metabolic process. The process itself is organized around a trigger word, often in the form of a verb, verb nominalization or adjective, e.g. *generates*, *hydroxylation, acetylated*.

The curators were asked to demarcate the metabolic process concepts and assign a corresponding semantic label, i.e. any of CC, GGP or trigger. As this task aims to ultimately facilitate the semi-automatic curation of biochemical reaction databases, it was desirable that the concepts be linked with entries in relevant biochemical resources. An additional requirement, therefore, was to assign to each concept a unique identifier from the following three vocabularies/ontologies: ChEBI (36) for CCs, UniProt (37) for GGPs and the CTD chemical–gene interaction ontology for trigger words.

To elucidate the specifications of the task and to foster agreement among annotators, we prepared a set of annotation guidelines describing the scope (i.e. what should be considered for marking up) and span (i.e. how to demarcate concepts within text) together with illustrative examples. The annotation guidelines, as well as the instructions, for using Argo for this particular task were made publicly available on Argo’s main website. They are also included as Supplementary Materials for the reader’s convenience.

### Annotation process

We randomly selected a subset of 60 PubMed abstracts from those that are tagged in the CTD as relevant to different types of metabolic processes. To draw parallels between purely manual and semi-automatic curation, we split the original data set into two equal parts and prepared a total of three workflows:

1. *Manual annotation*: This workflow reads PubMed abstracts and opens a Manual Annotation Editor for a curator to tag the entities of interest without any support from automatic processing available in Argo. It takes a list of PubMed abstract identifiers as input (by means of the PubMed Abstract Reader) and uses the XMI Writer to store the annotated documents in XMI format.
2. *Automatic annotation*: Purely automated and not involving any manual intervention from the curators, this workflow reads PubMed abstracts and performs recognition and normalization of the entities of interest. The objective of this workflow is to automatically ‘pre-annotate’ the input abstracts for later manual inspection (which for evaluation purposes is not part of this workflow). After fetching abstracts by means of the PubMed Abstract Reader, the workflow proceeds to the segmentation of the documents with the GENIA Sentence Splitter. This is followed by tokenization, part-of-speech and chunk tagging with the GENIA Tagger, which additionally performs recognition of GGPs. Chemical compounds and trigger words are then recognized by the OscarMER component, which is capable of recognizing six different types of chemically relevant concepts, including chemical molecules and reactions. An instance of the SPARQL Annotation Editor was introduced into the workflow to facilitate the transcription of the semantic types from the preceding recognizers into a unifying, compact and simplified annotation schema for future use by the curators. Automatic normalization (i.e. assignment of external resource identifiers) is then facilitated by the three linker components (ChEBI Linker, UniProt Linker and CTD Linker), which use the Jaro-Winkler string similarity algorithm (38) to match recognized concepts against entries in external databases/ontologies. Finally, the XMI Writer component saves the annotated abstracts in XMI format.
3. *Manual correction*: This workflow reads XMI files that already contain annotations (coming from the second automatic workflow) and opens a Manual Annotation Editor for a curator to correct (remove, add, modify) the automatically recognized annotations. The annotated files are saved in XMI format by the XMI Writer.

Although the second and third workflows could be combined into a single workflow, for the purposes of evaluation they were defined separately. We made the three workflows publicly available in Argo and accompanied them with detailed instructions available on the Argo website (see also Supplementary Materials).

For each PubMed abstract, a set of annotated text spans corresponding to GGPs, CCs and triggers were returned as output. The annotations for each text span include the location (i.e. document offsets), semantic label and the corresponding unique identifier from the relevant external resource. The annotations have been made available in the XMI, BioC and RDF formats with the aid of the various serialization components available in Argo (see Table 1).

### Curators

Together with the BioCreative track organizers, we recruited four curators with expertise in the curation of biochemical entities. One annotator was experienced in the semantic annotation of the PubChem databases (39). Two were scientific database curators for ChEBI, specializing in cheminformatics and metabolism. The last one was a curator for the MetaCyc database who, however, withdrew from the task during its early stages but managed to provide general feedback on the workbench.

## Results

We analyse the curation task quantitatively by computing effectiveness and agreement metrics over the sets of annotations produced by the curators, and qualitatively by reporting on the received feedback from the curators and the organizers.

One curator annotated mentions of all three concept types; the other two who specialize in ChEBI curation opted to focus only on chemical compound annotation. Consequently, we had at our disposal one set of abstracts with annotations for CCs, GGPs and triggers and two sets with only CC annotations.

### Suitability of automatic processing

In evaluating Argo in terms of the performance of its automatic processing, we compared the annotations generated by the ‘Automatic annotation’ workflow on the 60 PubMed abstracts against the curators’ annotations drawn from the outputs of the ‘Manual annotation’ and ‘Manual correction’ workflows, i.e. the curators’ annotations were treated like gold standard. A detailed breakdown on precision, recall and *F*-score for the different subsets and curators is given in Table 4. For GGPs and triggers (annotated by a single curator), the micro-averaged *F*-scores are at the level of 60 and 81%, respectively. For CCs, the *F*-score is 56% for one of the curators and rises to 74–75% for the other two. This discrepancy can be explained by looking into the scores for individual subsets. Whereas the performance of the automatic workflow is comparable among the three curators on the ‘manual annotation’ subset, it deviates significantly on the ‘manual correction” subset with the *F*-score values as high as 95%. This indicates that the presence of annotations affected the behaviour of the curators who gladly accepted most of the automatic propositions.

**Table 4.** The performance of the automatic workflows compared against the annotations of human curators

Curator	Category	Manual annotation			Manual correction			All		
		P	R	F	P	R	F	P	R	F
Curator 1	Chemicals	47	67	55	48	71	57	47	69	56
	GGPs	63	61	62	63	55	58	63	58	60
	Triggers	93	55	69	98	88	93	96	71	81
Curator 2	Chemicals	39	60	47	93	97	95	67	83	74
Curator 3	Chemicals	40	67	50	91	98	95	66	86	75
Majority voting	Chemicals	27	66	39	90	98	94	67	87	76
Union	Chemicals	57	49	53	82	96	89	72	73	72

The results are split into the two subsets of documents used in the curation task. P—precision, R—recall, F—*F*-score. Reported values are in percentages.

Table 4 also shows the performance of the automatic processing against the combined CC annotations (the only category annotated by more than one curator). We report on two ways for harmonizing the curators’ independent annotations: by majority voting, in which an annotation is retained only if at least two curators produced it, and by obtaining the union of all annotations. Overall, the precision–recall trade-off appeared to work well for the automatic processing, which achieved a higher *F*-score for the majority voting, albeit marginally, when compared with the individual scores.

### Inter-annotator agreement

The pair-wise inter-annotator agreement for CCs ranged between 67 and 84% in F-score with the two annotators specializing in CC achieving the upper bound. Table 5 shows the inter-annotator agreement in detail. The agreement rate on the first subset of documents varies slightly between the annotator pairs and shows fairly high level of agreement (76–82%), whereas the rate on the second ‘correction’ subset clearly confirms that two of the curators were mostly content with the automatic suggestions.

**Table 5.** The inter-annotator agreement

Curator	Manual annotation	Manual correction	All
Curator 1 and 2	76	57	67
Curator 2 and 3	76	92	84
Curator 1 and 3	82	56	69

The results are split into the two subsets of documents used in the curation task. Reported values are *F*-scores in percentages.

### Reconstructions of chemical–gene interactions in the CTD

We also analysed how well each of the curators as well as the automatic workflow reconstruct chemical–gene interactions that are stored in the CTD for each PubMed abstract. Each chemical–gene interaction in the CTD consists of the identifiers of the participants and the type of interaction. It must be noted, however, that the annotations produced by the human annotators and our automatic annotation workflow are not directly comparable with the CTD annotations for several reasons. While multiple instances of the same concept are contained in our corpus together with their in-text locations, the CTD stores only a per-document list of unique concepts without any location information. Additionally, we had to use approximate matching when mapping our GGPs and CCs to the identifiers of genes and chemicals in the CTD. In contrast, the interaction trigger words were mapped directly, as the task required the curators to link such words to entries in the CTD. Another source of incompatibility stems from the fact that for each abstract in the CTD, the database holds only identifiers of those chemicals and genes that directly participate in interactions mentioned in an abstract. Consequently, the list of curated CTD concepts might not contain all of the concepts mentioned in a document. In contrast, our task was designed to include the annotations of all of the mentions of chemicals, genes or gene products and metabolic process triggers. Table 6 summarizes the results of this comparison.

**Table 6.** The approximate and indirect mapping of human and automatic annotations to chemical–gene interactions in the CTD

Curator	Category	Manual annotation			Manual correction			All		
		P	R	F	P	R	F	P	R	F
Automatic workflow	Chemicals	16	55	25	16	49	24	16	52	25
	GGPs	14	37	21	14	34	19	14	36	20
	Triggers	63	42	50	53	51	52	58	46	51
Curator 1	Chemicals	33	68	44	32	64	43	32	66	44
	GGPs	20	40	26	18	39	24	19	39	25
	Triggers	55	58	57	46	60	52	51	59	54
Curator 2	Chemicals	31	67	43	18	53	27	24	60	34
Curator 3	Chemicals	36	66	46	17	49	25	24	58	34
Majority voting	Chemicals	35	67	46	18	52	27	25	60	35
Union	Chemicals	26	68	38	19	66	30	22	67	34

The results are split into the two subsets of documents used in the curation task. P—precision, R—recall, F—*F*-score. Reported values are in percentages.

As expected, the best results were obtained for trigger words, as they directly corresponded to the entries in the CTD. The only curator who produced trigger word annotations scored at the level of 54% in *F*-score, which is closely followed by the automatic workflow’s *F*-score at the level of 51%. The biggest differences can be observed among annotations of CCs where the aforementioned curator outperformed the other two as well as the automatic workflow by a 10%-point margin. However, the F-scores for the three curators are more balanced for the ‘manual annotation’ subset of abstracts. This shows yet again, that two of the curators could have been biased by the presence of automatic annotations.

### Curation time

The differences in the time spent on the task between the purely manual and the text-mining-assisted curations showed to be inconclusive. One of two curators who reported their times recorded a time reduction by a third with the text-mining-assisted set-up, whereas the other one reported an increase by a fifth. This inconsistency may be explained by the fact that the curators distributed their work over a month period, which may have affected their time keeping. We also note that the comparison baseline in our evaluation set-up was already demanding. Both the manual and assisted curation configurations featured the same manual annotation editor, which was virtually the only interface between the curators and Argo. It is a set-up that reaches far beyond the traditional curation process involving manually extracting and transferring relevant information from papers to spreadsheets. It is also worth noting that the time spent on automatic processing is only a fraction of the total time of the curation task. The initialization of resources, which is performed only once per the entire batch of documents, takes less than a minute, whereas the processing of a single document takes a few seconds.

### Qualitative analysis

The curators were also asked to provide feedback on various aspects of the system in the form of a survey carried out by the track organizers. The overall scores for experience, system rating and recommendation were high for two of the curators who scored the system 12 and 14 out of 15 and low for the other two, both rewarding only 5 points. Such polarized scores may be the result of the amount of time the curators dedicated to learn the system. The curators were asked to go through a series of tutorials to familiarize themselves with Argo before proceeding to the annotation task, which may have been neglected, e.g. one of the curators admitted to proceeding to the task without taking any training. We also decided to leave the configuration of workflows to the curators themselves to validate whether performing this task is suitable for users with a presumably limited technical background. The versatility and—inevitably—complexity of the system posed a barrier to the full use of the workbench for two curators.

To ease the use of Argo for curators, we have developed several new functionalities based on the received feedback. The biggest single inconvenience for the curators appeared to be the necessity of creating a workflow—albeit a simple one—to view or edit already saved manual annotations. We have tackled this problem by incorporating a document editing functionality directly in the Documents panel: users may now simply browse the documents in their space and open a selected one in the Manual Annotation Editor.

We also observed that some annotations made by curators included leading or trailing white-space characters (which were removed for the purpose of evaluation). To alleviate this issue, we introduced a feature in the Manual Annotation Editor that adjusts annotation spans to automatically exclude such characters. This, however, is made optional, as other annotation tasks may allow annotation boundaries to lie inside words. In fact, some of the annotations made by the curators span only a fragment of chemical expressions.

Other improvements included more convenient access to annotation guidelines during manual annotation process and several other graphical usability changes.

## Comparison with other systems

In this section, we present a short review of available Web-based biocuration platforms, conducted as a qualitative comparison of Argo against the other systems that participated in the interactive track of the BioCreative IV workshop.

Some of the participating systems, the CellFinder curation pipeline (40), BioQRator (41), RLIMS-P (42) and Egas (43), feature support for all the three broadly identified biocuration tasks, i.e. document selection, biomedical concept annotation and interaction annotation. The remaining systems are focussed on only one or two tasks: document selection in the case of SciKnowMine (44), concept annotation for MarkerRIF (45) and tagtog (46) and both concept and interaction annotation for ODIN (47). In comparison, Argo facilitates all the three tasks by means of its rich library of components and several improvements to the core system implemented to address the feedback received from the curators. For instance, the availability of the Kleio Search component allows users to supply a search query for selecting only documents of particular relevance. If further triage is needed, users may opt to save the search results in their storage space and manually sieve the documents by viewing them in the built-in annotation editor and deleting irrelevant items. Argo provides even stronger support for the two latter biocuration tasks with its wide range of available options for bioconcept recognition and interaction extraction (shown in Table 2).

Most of the participating systems were tailor-made for specific subject domains, therefore restricting their applicability to other biocuration tasks. The CellFinder curator and RLIMS-P systems are both tightly coupled with task-specific text-mining pipelines, requiring significant effort in adapting them to new curation projects. The named entity recognition and event extraction tools used by the CellFinder curator, for instance, were based on dictionaries and statistical models focussed on cells and genes. Similarly, RLIMS-P was developed as a rule-based system particularly for extracting phosphorylation events involving proteins acting as kinases, substrates and sites. Although both ODIN and BioQRator are also closely tied to specific tools [i.e. OntoGene (48) and PIE the Search (49), respectively], they claim to be curation interfaces that can be customized via integration with other text-mining pipelines. Customization is supported to a slightly greater extent by Egas and tagtog, which allow for the creation of configurable projects with custom annotation types. Likewise, SciKnowMine facilitates text-mining-assisted document triage for any subject domain with its support for offline training of triage models on administrator-specified data sets. This is also the case for semi-automatic concept annotation in tagtog, in which models can be developed based on custom dictionaries and user-provided annotation input (i.e. in an active learning-like manner). In terms of task and domain adaptability, Argo is much more advanced in its flexibility of allowing annotation project proponents to define workflows and select underlying text-mining components according to the requirements of a task at hand.

To a certain extent, some of the aforementioned biocuration systems render support for interoperability with external tools. Driven by text-mining components in the form of RESTful Web services, MarkerRIF and Egas can be integrated with other tools implemented in the same standard. BioQRator and Egas support the storage of annotations in the BioC format, allowing other BioC-compliant systems to read and process the resulting data. In comparison, Argo advances interoperability to a much greater extent by the availability of data serialization and deserialization components supporting exchange format standards such as XMI, RDF and BioC. Argo also features RESTful versions of these components, which makes it possible to deploy workflows as Web services.

## Conclusions

Primarily a text-mining workbench, Argo features a number of capabilities that render the system suitable for performing curation tasks. Given the nature of the system, Argo is best fitted for tasks related to the extraction of information from data (such as relevant concept recognition and concept interaction identification), but also exhibits strong potential in supporting document triage, as demonstrated by the use of a search engine reader component.

The curation task carried out for the purpose of evaluating the workbench showed that the introduction of automatically generated annotations, which were meant to support the manual curation effort, tend to influence the decision of curators. Harmonized and impartial results could be obtained by the active collaboration of all curators involved in an annotation effort, as well as the automatic processing to speed up the task. Such active collaboration is already possible in Argo and is realized by means of the Manual Annotation Editor. The editor allows several users to remotely work on the same document (or a set of documents) and see each other’s changes in their respective instances of the editor in real time.

Argo may be used by biocuration teams as a one-stop solution without any additional tool-development efforts; however, this type of use has its limitations. The current state of Argo will appeal to teams that are happy with working with their data through the functionality provided by the Documents panel and the currently available components facilitating document triage and curation result serialization. Further integration with certain teams’ existing infrastructure (particularly databases) can be achieved by the development of dedicated components that can be included in Argo workflows by the use of the Generic Listener component, which may be used to, for example, save results in a specific remote database. This type of integration, however, does require some development effort. To accelerate such efforts, a software development kit is provided together with sample code.

Argo has a clear advantage over other systems in terms of the flexibility of defining biocuration tasks. The versatility of the system, however, comes with a complexity that impedes its usability when compared with other solutions. The evaluation of the workbench by biocurators participating in the BioCreative IV challenge revealed that two of the four curators struggled with the task when attempting it without prior training. In contrast, the other two curators who underwent the training rated the system highly.

Drawing from the received feedback, we identified areas of improvement. In particular, we have already addressed the issue of directly editing annotations in users’ documents without the need to build a workflow, thus shifting the workflow-centric nature of Argo to a document-centric one. We would also like to explore its already available but underexploited resource sharing capabilities. Specifically, we are planning to draw a clear distinction between capabilities designed for technical users who are adept at building workflows, and users who have the domain expertize needed for validating the results of the processing of such workflows.
